# *STXBP2-R190C* Variant in a Patient With Neonatal Hemophagocytic Lymphohistiocytosis (HLH) and *G6PD* Deficiency Reveals a Critical Role of STXBP2 Domain 2 on Granule Exocytosis

**DOI:** 10.3389/fimmu.2020.545414

**Published:** 2020-10-08

**Authors:** Nathalia Benavides, Waldo A. Spessott, Maria L. Sanmillan, Marcelo Vargas, Mylynda S. Livingston, Nissa Erickson, Tamara C. Pozos, Margaret E. McCormick, Emilia Scharrig, Yoav H. Messinger, Claudio G. Giraudo

**Affiliations:** ^1^Department of Microbiology and Immunology- Sydney Kimmel Medical College- Thomas Jefferson University, Philadelphia, PA, United States; ^2^Department of Medical Genetics and Genomics, Children's Minnesota, Minneapolis, MN, United States; ^3^Department of Pediatric Hematology–Oncology, Children's Minnesota, Minneapolis, MN, United States; ^4^Minnesota Gastroenterology, P.A., Minneapolis, MN, United States; ^5^Department of Immunology, Children's Minnesota, Minneapolis, MN, United States

**Keywords:** familial hemophagocytic lymphohistiocytosis−5 (f-HLH), STXBP2, G6PD deficiency, cytotoxic T-lymphocytes (CTLs), natural killer cells, lytic granule exocytosis, SNAREs

## Abstract

Neonatal hemophagocytic lymphohistiocytosis (HLH) is a medical emergency that can be associated with significant morbidity and mortality. Often these patients present with familial HLH (f-HLH), which is caused by gene mutations interfering with the cytolytic pathway of cytotoxic T-lymphocytes (CTLs) and natural killer cells. Here we describe a male newborn who met the HLH diagnostic criteria, presented with profound cholestasis, and carried a maternally inherited heterozygous mutation in *syntaxin-binding protein-2* [*STXBP2*, c.568C>T (p.Arg190Cys)] in addition to a severe pathogenic variant in *glucose 6-phosphate dehydrogenase* [*G6PD*, hemizygous c.1153T>C (Cys385Arg)]. Although mutations in *STXBP2* gene are associated with f-HLH type 5, the clinical and biological relevance of the p.Arg190Cys mutation identified in this patient was uncertain. To assess its role in disease pathogenesis, we performed functional assays and biochemical and microscopic studies. We found that p.Arg190Cys mutation did not alter the expression or subcellular localization of STXBP2 or STX11, neither impaired the STXBP2/STX11 interaction. In contrast, forced expression of the mutated protein into normal CTLs strongly inhibited degranulation and reduced the cytolytic activity outcompeting the effect of endogenous wild-type STXBP2. Interestingly, arginine 190 is located in a structurally conserved region of STXBP2 where other f-HLH-5 mutations have been identified. Collectively, data strongly suggest that STXBP2-R190C is a deleterious variant that may act in a dominant-negative manner by probably stabilizing non-productive interactions between STXBP2/STX11 complex and other still unknown factors such as the membrane surface or Munc13-4 protein and thus impairing the release of cytolytic granules. In addition to the contribution of STXBP2-R190C to f-HLH, the accompanied *G6PD* mutation may have compounded the clinical symptoms; however, the extent by which *G6PD* deficiency has contributed to HLH in our patient remains unclear.

## Introduction

Neonatal hemophagocytic lymphohistiocytosis (HLH) is a medical emergency that can be associated with significant morbidity and mortality. Patients can present a diagnostic dilemma, especially when the presentation is deemed atypical (1, 2). Newborns and infants manifesting with HLH often have a genetic determined HLH, also known as familial HLH (f-HLH) (3–5). f-HLH is caused by autosomal-recessive gene mutations that impair lymphocyte cytotoxicity such as *PRF1*, (FHL-2) (6), *UNC13D* (FHL-3) (7), *STX11* (FHL-4) (8), *STXBP2* (FHL-5) (9, 10), and *RAB27A* (Griscelli syndrome II) (11). However, when the functional consequence of a mutation in any of these f-HLH genes is unclear, it further confuses the clinical picture and can lead to delay in therapy. f-HLH was initially described in patients as a consequence of monogenic autosomal-recessive mutations. Nonetheless, the landscape of genetic mutations underlying pediatric f-HLH has further expanded, and it has also been associated with heterozygous mutations in f-HLH genes, either as monogenic or digenic inheritance, as well as with mutations that can act in a dominant-negative fashion (12–15).

Over the last years, several mutations in *STXBP2* gene have been identified in f-HLH-5 patients manifesting with variable clinical presentations (9, 10, 16, 17). However, for many of these mutations, it is still not clear how they impact on the molecular mechanism of cytotoxic granule secretion. *STXBP2* gene encodes for the protein Munc18-2 that belongs to the Sec/MUNC (SM) protein family. SM proteins are essential components of multiple intracellular membrane trafficking steps in eukaryotic cells (18, 19). They function along with the universal membrane fusion machinery, soluble N-ethylmaleimide–sensitive factor attachment protein receptors (SNAREs), to ensure specificity, and control lipid membrane fusion. SM proteins interact with SNAREs in multiple ways using their central cavity and other domains. They can bind monomeric t-SNAREs, for example, STX11, as well as assembled SNARE complexes composed of STX11/SNAP23/VAMP8 (20–23). Varying functions have been attributed to the different binding modes of MUNC18s with SNARE proteins. For example, MUNC18-2 can operate as a chaperone of monomeric STX11 facilitating transport to its final destination (at the plasma membrane), as well as an activator for membrane fusion by promoting SNARE complex assembly (21, 23). However, how mutations in STXBP2 associated with f-HLH interfere with different functions of Munc18-2 has remained poorly understood.

Here, we describe a male newborn with neonatal HLH carrying a maternally inherited monoallelic mutation in *STXBP2*, c.568C>T (p.Arg190Cys), which is also present in two of the four siblings and a severe pathogenic variant in G6PD [hemizygous c.2T>C (p.Cys385Arg)]. Functional CD107a degranulation assays showed that natural killer (NK) cells and CD8^+^ lymphocytes from patient, mother, and carrier siblings displayed a severe reduction in their ability to degranulate. Cell killing assays showed a partial reduction in the cytotoxic capacity of patient CD8^+^ cells against target cells. Biochemical analysis shows that mutation R190C in *STXBP2* does not seem to disrupt protein or *m*RNA stability because it does not affect the STXBP2 protein expression levels in patient's peripheral blood mononuclear cells (PBMCs), neither its interaction with endogenous syntaxin-11 (STX11). The forced expression of STXBP2-R190C in healthy control (H.C.) CD8^+^ cells resulted in a severe impairment of granule exocytosis evidenced by CD107a degranulation and cell-mediated cytotoxicity. Our results indicate that mutation R190C has a severe deleterious effect on STXBP2 function and thus in lytic granule secretion. Interestingly, Arg190 residue is located in a region of domain 2 where other *STXBP2* mutations have been found in f-HLH patients (15, 17). Because this region is highly conserved in both protein sequence and three-dimensional structure, these results suggest that this undiscovered region of STXBP2 may play a critical role during lytic granule exocytosis in CD8^+^ and NK cells. Taken together, this study shows that mutation R190C in STXBP2 impairs protein function in a dominant-negative fashion that individuals carrying the mutation STXBP2-R190C display an abnormal CD8 and NK cell cytotoxic function and that the accompanied *G6PD* mutation may compound the clinical symptoms and thus facilitate the triggering HLH.

## Materials and Methods

### Case Presentation

A term Caucasian male was born via normal spontaneous vaginal delivery and a birth weight of 3.6 kg. He presented at 8 h a profound conjugated hyperbilirubinemia (bilirubin total/direct 32.9/24.0 mg/dL). Family history of G6PD deficiency, elevated reticulocytes, and high lactate dehydrogenase (LDH) were suggestive of hemolysis, but Heinz bodies prep was negative. Thrombocytopenia markedly elevated ferritin (20,365 ng/mL), hepatosplenomegaly (HSM), liver dysfunction, and elevated soluble interleukin 2 (IL-2) receptor were suggestive of HLH (Table 1). CD107a degranulation was decreased; bone marrow showed hemophagocytosis, and liver biopsy showed a dense histiocytic infiltrate in a background of neonatal hepatitis, consistent with HLH. Lymphocyte phenotyping showed normal numbers of CD3 T cells and no increase in activated T cells. Expression of SAP and XIAP in CD8^+^ T cells and NK cells was normal, ruling out X-linked lymphoproliferative disease. As noted by Allen et al., the marked elevated ferritin of >10,000 in pediatrics is highly diagnostic of HLH (24). Certainly, the clinical picture with hemophagocytosis in the bone marrow biopsy and decreased CD107a are also suggestive that this is a true causative effect (25). It may be relevant that NK cytotoxic activity was partially reduced, but CD107a degranulation is abnormal, as has been reported for patients with splicing mutations in *STXBP2* associated with immunodeficiency and late-onset HLH (26). Although it is atypical for classic cases of primary HLH, the percentage of activated T cells was normal as it was previously observed in other cases (27).

**Table 1:** HLH-related laboratory data.

	**Lab reference**	**Day 1**	**Day 12**	**Day 38 initiate chemo**	**Day 101 cont therapy**	**Day 172 24 weeks**	**Day 321 46 weeks complete therapy**
Platelets, /μL	150,000–450,000	65,000	161,000	889,000	485,000	519,000	511,00
Hgb, g/dL	9.5–13.5	15.6	14.5	11.0	9.6	13.0	7.1
ANC, /μL	1,000–8,000	3,700	7,500	7,500	1,500	2,580	2,800
Total bilirubin, mg/dL	0.2–0.9	32.4	11.4	6.2	0.4	0.7	0.5
Direct bilirubin, mg/dL	0.0–0.3	27.2	9.7	4.8	NA	0.1	
ALT, U/L	6–50	507	127	622	66	41	32
AST, U/L	10–74	1,924	406	411	53	99	43
LDH, U/L	250–568	3,977		295		674	
Reticulocyte, %	0.99–1.82%	20%				1.6%	
Ferritin, ng/mL	50–200	20,365	4,930	4,091	1,717	865	233
Fibrinogen, mg/dL	200–400	199	318			216	
Soluble interleukin-2 receptor, pg/mL	≤1,033	1,206				1,194	
NK function lytic units^*^	>=2.6	21.9 (normal)					
CD107a degranulation^**^				Reduced			

* *NK function was obtained on day 5*.

** *CD107 day of life 24−3% (11–35), MCF 33 (207–678)—decreased CD107a expression*.

Gestational alloimmune liver disease (GALD) was unlikely as there was no maternal history of miscarriage or infant loss, and he had normal abdominal magnetic resonance imaging and normal buccal biopsy. No bacterial or viral pathogens were found—no metabolic disorders and no mitochondrial disorders or bile-acid synthetics abnormalities.

With suspected GALD, he received intravenous immunoglobulin (IVIG) (1 g/kg per dose, 3.5 g total) on days of life (DOL) 1–5 that resulted in improved liver functions and ferritin and normalization of the platelet count (Table 1). However, liver transaminases remained moderately elevated, suggesting the need for additional therapy. Therefore, on DOL 12, a pulse of dexamethasone 10 mg/m^2^ daily was given × 14 days. Transaminases and ferritin remained elevated, resulting in initiation of dexamethasone, etoposide, and monthly IVIG on DOL 38 (age 5 weeks). This led to complete normalization of transaminases, and the HSM also resolved. Because ferritin remained elevated by DOL 101 (age 14.5 weeks), continuation therapy with pulses of dexamethasone, etoposide, and cyclosporin-A (CsA) was given. Treatment was complicated by significant anemia requiring packed red blood cell (pRBC) transfusions and decreased etoposide dosing. With suspected CsA-induced eryptosis, CsA was weaned slowly from age 34 weeks. All therapy was discontinued at age 46 weeks. He is currently 2.5 years old growing and thriving with no evidence of HLH reactivation. After discontinuing IVIG, from the age of 1.5 years, he did suffer four episodes of viral-induced hemolysis that were attributed to his severe G6PD deficiency requiring blood transfusion with each episode. No reactivation of HLH was noted.

### Antibodies

Mouse anti-CD3 (OKT3 functional grade purified) was purchased from BD Pharmingen (San Jose, CA, USA). Rabbit anti–syntaxin 11 and rabbit anti-STXBP2 were purchased for Synaptic System (Göttingen, Germany). Mouse anti–MUNC13-4 was purchased from Santa Cruz Biotechnology (Dallas, Texas, USA). Rabbit anti–F-actin was purchased from Sigma (St. Louis, MO, USA). Secondary antibodies donkey anti–rabbit-IRDye800 CW-conjugated and donkey anti–mouse-IRDye680-RD conjugated were purchased from LiCor (Lincoln, NB, USA).

### Cells and Transfection

Written consent was obtained from the family of the f-HLH-5 patient using a protocol approved by the institutional review board at Minnesota Children's Hospital. Control blood samples were collected in EDTA tubes and processed within 24 h of venipuncture. PBMCs were obtained by density gradient centrifugation (Lymphoprep, Axis-Shield) and resuspended in complete medium (RPMI 2 supplemented with 10% fetal bovine serum, l-glutamine, penicillin, and streptomycin; all from Invitrogen). Isolated cells were activated and expanded using Dynabeads (Human T-Expander CD3/CD28 from Life Technologies; Grand Island, NY, USA) for 5 days in complete medium. After this time, beads were removed using a magnet and cells used for different experiments. The human K562 erythroleukemia and murine P815 mastocytoma cell line were from the American Type Culture Collection (Manassas, USA) and maintained in complete medium.

CD8 cells from H.C. were transfected with the indicated ECFP-STXBP2 constructs using the NEON microporation system (Invitrogen, Grand Island, NY, USA) using a 100 μL tip and following manufacture's protocol for PBMC transfection.

### Cytotoxicity Assays

The killing activity of effector cells was evaluated using a non-radioactive cytotoxicity assay Cytotox-96 (Promega) following the manufacturer's specifications. For assessment of cytotoxicity, expanded PBMCs were supplemented with 0.5 μg/mL anti-CD3 mAb, mixed with 2 × 10^4^ target P815 cells and incubated in quadruplicate for 4 h at 37°C. Effector-to-target cell ratios ranged from 10 to 0.65 in 100 μL medium in 96-well V-bottom plates. After 4 h, plates were centrifuged at 250 × g for 4 min, and 50 μL of supernatant was transferred to a new flat-bottom 96-well-plate; 50 μL of the substrate was added to each well in the new plate and incubated for 30 min at room temperature. The reaction was stopped using 50 μL of stop solution for each well. LDH release was measured at 490 nm using a 96-well-spectrophotometer (Spectramax, Molecular Devices). The following formula was used to calculate the percent cytotoxicity: cytotoxicity (%) = (experiment – effector spontaneous – target spontaneous/target maximum – target spontaneous) × 100. Also, the killing activity of the patient CD8^+^ cells was evaluated with the EarlyTox Caspase-3/7 NucView 488 Assay Kit (Molecular Devices) following manufacturer's instructions. Briefly, target and effectors cells were incubated separately with a membrane-permeable NucView-488 caspase 3/7 substrate during 30 min. CD8^+^ cells were activated with anti-CD3 (OKT3) antibody 15-min previous incubation with the substrate ends. Effectors and target cells were combined in 10:1 ratio in a black flat-bottom 96-well-plate. The fluorescence was measured during 130 min with an interval of 5 min in a fluorescence microplate reader (ImageXpress® Pico–Molecular Devices). The percentage of specific cell death was measured a percentage of cells that became positive for NucView 488 signal over the time.

### Degranulation Assay

PBMCs isolated from control and patient samples were incubated in the absence or presence of target cells (K562 or P815) at 1:1 ratio for 2 h at 37°C. After this time, cells were labeled using the following combination of fluorescently labeled antibodies: anti–CD107a-PE, anti–CD56-APC, anti–CD8-FITC, and anti–CD3-PerCP. Data were acquired using LSR-II flow cytometer (BD) and with CytoFlex-S (Beckman). For NK cell analysis, CD3^−^CD56^+^ NK cells were gated and assessed for surface expression of CD107a. For CD8^+^ cell analysis, CD3^+^CD8^+^ cells were gated and assessed for surface expression of CD107a. The term “% CD107a appearance” reflects the difference between the percentage of NK cells expressing surface CD107a after target cell stimulation and the percentage of NK/CD8^+^ cells expressing surface CD107a after incubation with medium alone.

### Coimmunoprecipitation Assays

Primary CD8^+^ cells were lysed using a lysis buffer (25 mM Tris, 150 mM NaCl, 1 mM EDTA, 5% glycerol; pH 7.4) containing 1% NP-40 followed by centrifugation at 14,000 rpm for 15 min. Coimmunoprecipitation experiments were carried out using the CO-IP kit from Pierce (Thermo Scientific) following manufacturer's instructions. Briefly, after cell lysis and centrifugation, supernatant was collected and incubated with agarose beads coupled with anti-STXBP2 antibody for 20 h at 4°C. Complexes bound to the beads were pelleted by centrifugation followed by five washes with lysis buffer containing 1% NP-40. Proteins bound to the antibodies were eluted using elution buffer pH 2.8. The eluted fractions were tested by Western blotting using the indicated antibodies.

### Immunostaining and STED Microscopy

CD3/CD28-activated cytotoxic T-lymphocytes (CTLs) from H.C. individuals were transfected with the indicated constructs using a Neon Microporation System (Thermo Scientific) and mixed with or without of P815 target cells at 1:1 ratio, pelleted by centrifugation, and incubated for 15 min to generated conjugates. Cells were then seeded in 24-well-plates containing polylysine-coated glass cover slips. Cells were fixed for 10 min with PBS containing 4% paraformaldehyde. Cells were permeabilized with PBS buffer containing 0.1% Triton X-100 and then blocked with 3% bovine serum albumin in PBS for 30 min at room temperature. Cover slips were extensively washed with PBS and mounted with Prolong Gold antifade reagent (Invitrogen). Images were collected using a Leica SP8-STED-3X microscope (Leica Microsystem) using cyan fluorescent protein and yellow fluorescent protein settings.

## Results

### Subjects Carrying STXBP2 R190C Variant Display Impaired CTL and NK Cell Function

**Figure 1:**
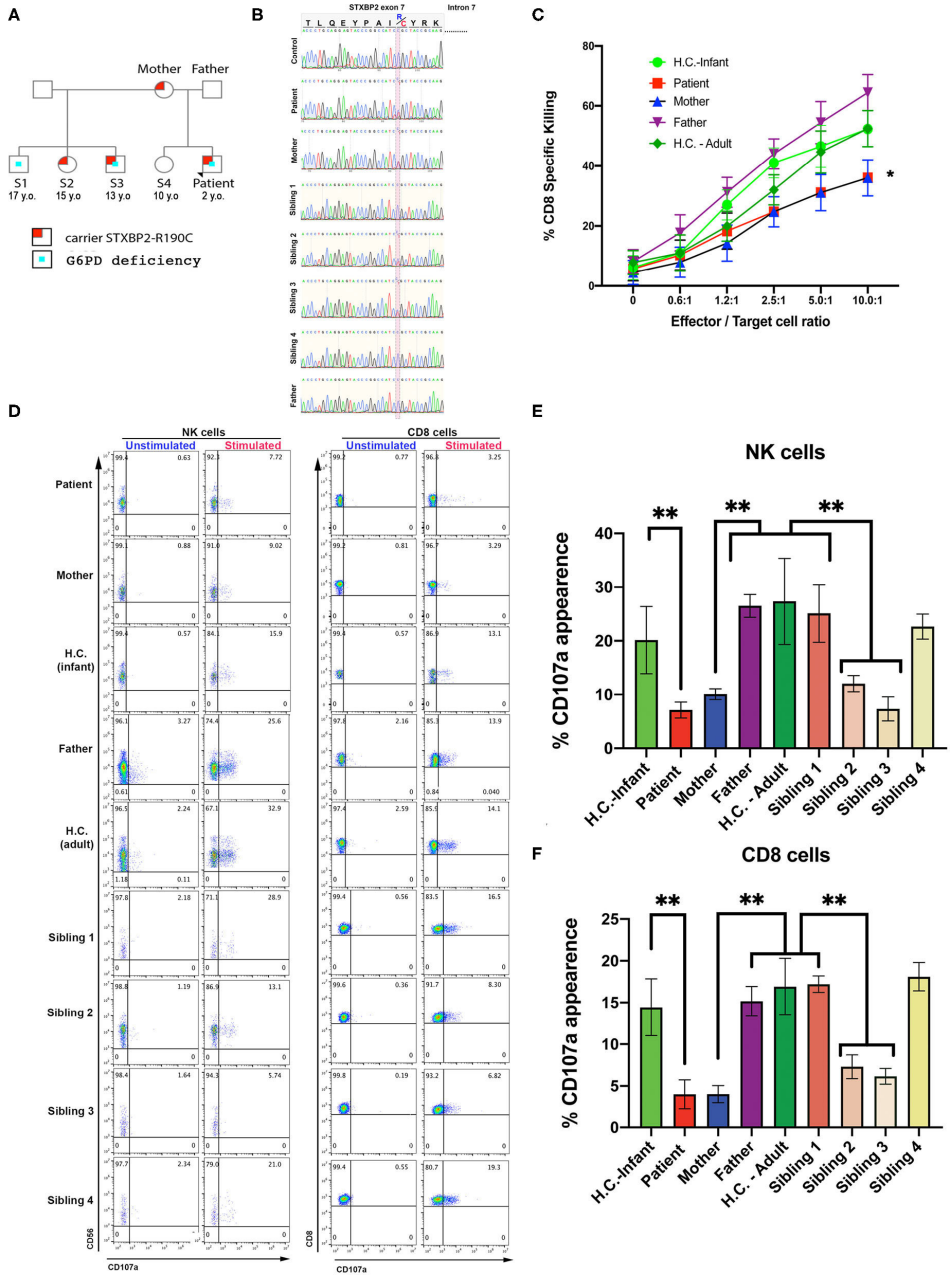
CTL and NK cells expressing the *STXBP2*^R190C^ mutation exhibit impaired functions. **(A)** Pedigree showing the inheritance of genetic variant and clinical traits. **(B)** Patient carries a maternally inherited heterozygous R190C variant. Sanger sequencing chromatograms of the genomic DNA region of STXBP2 extending from exon 7 and intron 7 of a healthy control (H.C.), patient and mother. Patient and mother have one allele with C > T variant (yellow shaded) that results in arginine-to-cysteine substitution. **(C)** Cytotoxicity assay to measure CTL-mediated cell killing. Equivalent numbers of CD8^+^ T cells (effectors) from age-matched H.C. (infants: green circles; adults: green diamonds), patient cells (red), mother (blue), and father (purple) were incubated with anti-CD3 antibody in the presence or absence of P815 target cells (targets) at the indicated cell ratios. **(D)** CD107a assay to measure degranulation of NK and CD8 cells. PBMCs from age-matched H.C., patient, mother, and father were incubated in the presence or absence of K562 cells (for NK cells assay) or P815 and anti-CD3 antibody (for CD8 assay) for 4 h at 37°C. Cells were stained using anti–CD107a-PE, anti–CD56-APC, anti–CD8-FITC, and anti–CD3-PerCP antibodies and analyzed by flow cytometry. CD3^−^CD8^−^CD56^+^ cells were gated for NK cells and CD3^+^CD8^+^ for CD8 cells and analyzed for the appearance of CD107a on the surface upon incubation with target cells. **(E,F)** Graphs showing the percentage of NK cells **(E)** and CD8 cells **(F)** that increased CD107a staining upon stimulation. Plots are mean ± S.D. of 3–4 independent experiments. Statistical analysis was done by one-way ANOVA, **p < 0.01. **(E,F)** Graphs showing the percentage of NK cells **(E)** and CD8 cells **(F)** that increased CD107a staining upon stimulation. Plots are mean ± S.D. of 3–4 independent experiments. Statistical analysis was done by one-way ANOVA ** p<0.01.

A term Caucasian male born via normal delivery presented within 8 h of age with hypoglycemia, profound conjugated hyperbilirubinemia, thrombocytopenia, and markedly elevated ferritin. He displayed HSM, liver dysfunction, and elevated soluble IL-2 receptor. Bone marrow showed hemophagocytosis, and liver biopsy showed a dense histiocytic infiltrate in a background of neonatal hepatitis, consistent with HLH (see *Materials and Methods, Case Report*, and Table 1). An HLH-gene panel exome sequencing revealed a heterozygous variant in exon 7 of *STXBP2*, c.568C>T (p.Arg190Cys) with uncertain clinical significance but predictive to be pathogenic by *in silico* analysis (polyphen and SIFT). *STXBP2* has been associated with autosomal recessive familial-HLH type 5 (f-HLH-5, Online Mendelian Inheritance of Man # 217). No other sequencing or copy number variants were found in additional genes tested [*AP3B1, BLOC1S6* (*PLDN*), *TNFRSF7* (*CD27*), *UNC13D* (*MUNC13-4*), *XIAP* (*BIRC4*), *ITK, LYST, MAGT1, PRF1, RAB27A, PRF1, SH2D1A, SLC7A7, STX11*]. Sanger sequencing was performed on the region between exon and intron 7 of patient, mother, sibling, half-siblings, father, and H.C. to confirm the identified variant and mode of inheritance. Results showed that patient, mother, and two half-siblings (S2, S3), but not S1, S4, father, and H.C., carry the same variant *STXBP2*, c.568C>T (p.Arg190Cys) (Figures 1A,B).

Additionally, whole-genome sequencing studies were performed on a research basis with corresponding parental consent. Patient was found to have a pathogenic variant in *glucose 6-phosphate dehydrogenase* (*G6PD*) [heterozygous c.2T>C (Cys385Arg) dbSNP:rs222 Tomah variant, WHO type I variant <10% of normal G6PD activity]. Consequently, he was also diagnosed with severe *G6PD* deficiency as his two other male half-siblings (S1, S3). Interestingly, however, he only had evidence of transient hemolysis before he was started on CsA treatment.

CTL and NK cell functions were assessed using PBMCs from the patient, mother, father, and H.C. age-matched (H.C.–infant; H.C.–adult) as previously described (14). The cytotoxic activity of CD8^+^ T cells was determined toward P815 target cells incubated in the presence of anti-CD3 antibodies. Cytolytic activity was tested in quadruplicate at different effector/target (E:T) cell ratios ranging from 10:1 to 0.65:1. These assays revealed that the CD8^+^-specific killing activity of the patient and mother was ~20–35% reduced compared with that of control CD8^+^ T cells (Figure 1C). Given the partial reduction in lytic activity, we next determined whether this defect was due to impaired secretion of lytic granules. Toward this end, we measured NK cell degranulation upon exposure to K562 target cells (28, 29). In these assays, we observed that significantly fewer NK cells of the patient (7.7%), mother (9.0%), sibling 2 (13.1%), and sibling 3 (5.4%) displayed CD107a at the cell surface compared with NK cells from father (25.6%), H.C.–infants (15.9%), sibling 1 (28.9%), sibling 4 (21.0%), or H.C.–adults (32.9%) (Figures 1D left panel, E). A similar CD107a degranulation defect was also evidenced in CD8^+^ cells (Figures 1D right panel, F). Taken together, these results demonstrate that the STXBP2 R190C mutation partially impairs effector cell cytolytic function but severely compromised lytic granule release in a manner similar as in the f-HLH-5 disorder.

### The R190C Mutation Does Not Affect STXBP2, STX11, or MUNC13-4 Protein Expression Levels

**Figure 2:**
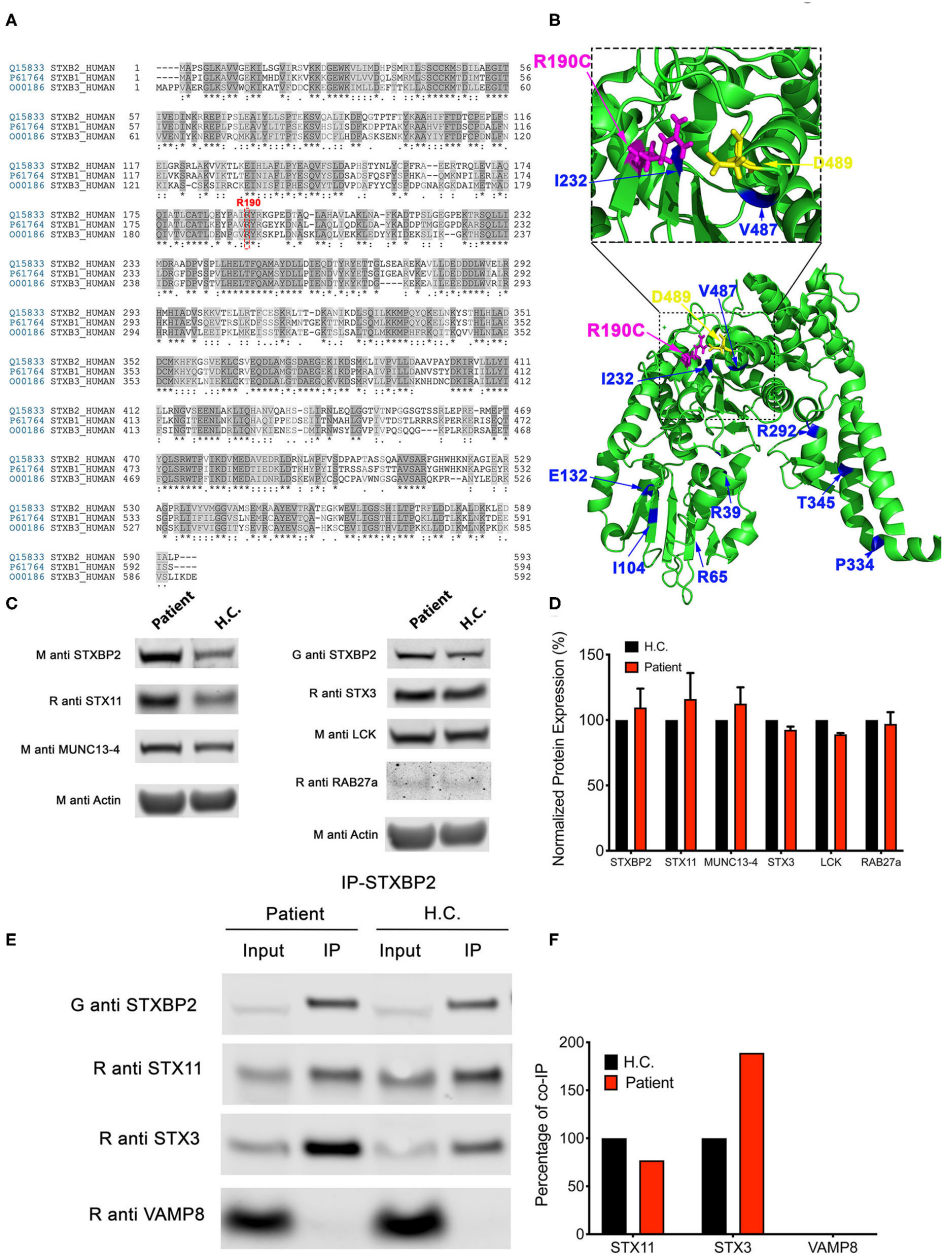
The *STXBP2* mutation does not influence protein expression or the Munc18-2/STX11 interaction. **(A)** Alignment of human STXBP1/2/3 showing that arginine 190 reside is conserved among them. **(B)** Crystal structure of Munc18-2 (PDB:4CCA) with the R190 residue highlighted in magenta, residue D489, which makes electrostatic interaction with R190, in yellow and previously described f-HLH-5 mutations are shown in blue. **(C)** Western blots showing the expression levels of Munc18-2, STX11, STX3, MUNC13-4, RAB27a, and Lck in lysates prepared using PBMCs from H.C. and patient. Actin staining of the same membranes was used to assess for equivalent protein loading. **(D)** Bands in the Western blot that corresponded to Munc18-2, STX11, and MUNC13-4 were quantified by densitometry and normalized to the intensity of actin in the same lane. Densitometry results are expressed as the percentage of those obtained using control samples, which were set as 100%. Values represent the mean ± SD of two independent experiments. **(E)** Coimmunoprecipitation experiments using lysates generated from H.C. or patient PBMCs. Endogenous STXBP2 was immunoprecipitated using an anti–STXBP2 antibody, and the amount of STX11, STX3, and VAMP8 that coimmunoprecipitated was analyzed by Western blotting. **(F)** Bands in the Western blot that corresponded to the fraction of STX11, STX3, and VAMP8 that coprecipitated with STXBP2 were quantified by densitometry and normalized to the amount of STXBP2 immunoprecipitated in the same lane. Densitometry results were expressed as the percentage of those obtained in control samples, which were set as 100%.

Arginine 190 resides within a region of domain 2 highly conserved among different SM proteins (STXBPs 1-3; Figure 2A). The crystal structure of STXBP2 shows that R190 is located closely to two previously described mutations associated with f-HLH: V487M (15) and I232del (10, 17) (Figure 2B). Interestingly, V487 is next to D489 that forms an ionic bond with R190. Some f-HLH-5 patients bearing mutations in *STXBP2* exhibit low levels of STXBP2 protein, probably due to mRNA or protein instability (10). As STXBP2 binds to and stabilizes STX11, these patients usually also show low STX11 levels (9, 10, 16). To elucidate the cause for the reduced cytotoxicity and degranulation of this patient's CTL and NK cells, we evaluated the protein expression levels of STXBP2 and STX11, as well as of other related f-HLH protein MUNC13-4, STX3, and Lck. For this purpose, equivalent amounts PBMC lysates from H.C. individuals or the patient were analyzed by Western blotting. Results showed that STXBP2, STX11, MUNC13-4, STX3, RAB27a, and Lck were expressed at similar levels in H.C. and patient cells (Figure 2C). The intensity of the bands corresponding to each protein was quantified by densitometry and normalized to actin as a loading control. This analysis showed comparable values between the patient and control samples (Figure 2D). These results indicate that the impaired cytotoxic activity of the immune cells in this patient was not due to decreased expression of STXBP2, STX11, RAB27a, or MUNC13-4.

To further evaluate whether the R190C mutation affects the ability of STXBP2 to bind to STX11, we performed coimmunoprecipitation experiments in which endogenous STXBP2 in PBMCs was immunoprecipitated and the amount of associated STX11 was analyzed by Western blotting. We observed that the amount of STXBP2 immunoprecipitated with an anti-STXBP2 antibody was comparable in the patient and control samples, as it was the amount of STX11 that coimmunoprecipitated with STXBP2 (Figure 2E, IP lane). Densitometry analysis of the STX11 bands normalized to the amount of immunoprecipitated STXBP2 further supported these results (Figure 2F). Similarly, STX3, a known interactor of STXBP2 (21), but not Vamp8, coimmunoprecipitated with STXBP2 to the same extent in patient and H.C. samples.

### STXBP2-R190C Displays a Normal Localization Pattern

**Figure 3:**
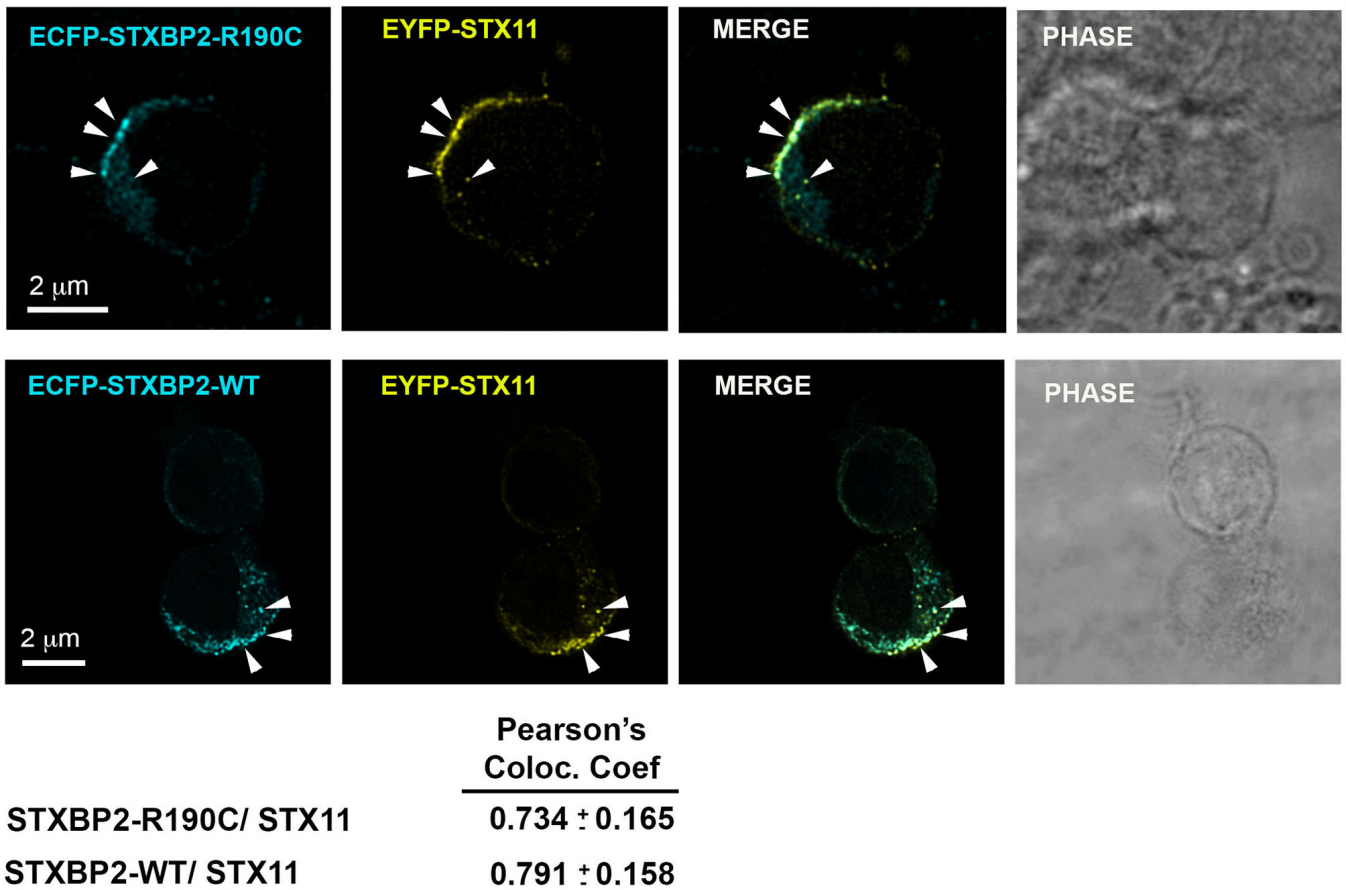
The R190C mutation does not affect the subcellular localization of STX11. CTLs from an H.C. individual were cotransfected with EYFP-STX11 and either ECFP-STXBP2-WT or ECFP-SXBP2-R190C; cells were incubated in the presence or absence of anti-CD3 coated P815 cells at a 1:1 ratio for 15 min at 37°C on polylysine-coated coverslips. Cells were fixed, mounted and imaged. Bars equal 2 μm. Pearson colocalization coefficient between ECFP-STXBP2-WT or R190C and EYFP-STX11. Values represent the mean ± SD; *n* = 15 cells.

STXBP2 was proposed to function as a chaperone by binding monomeric STX11 and facilitating its transport to the plasma membrane (21, 22). To determine whether STXBP2-R190C interferes with this function, we analyzed the subcellular localization of EYFP-STX11 when it is cotransfected with either ECFP-STXBP2-WT or ECFP-STXBP2-R190C in human CD8 lymphocytes. Results showed that EYFP-STX11 localized to intracellular vesicles and on the plasma membrane at the immunological synapse (Figure 3, arrowheads) in both ECFP-STXBP2-WT– and -R190C–cotransfected cells. The extent of colocalization between EYFP-STX11 with either ECFP-STXBP2-WT or ECFP-STXBP2-R190C was similar, as evidenced by the Pearson colocalization coefficient (Figure 3). These results suggest that the mutation R190C in STXBP2 does not interfere with the interaction with STX11 and neither with its subcellular distribution.

### Expression of STXBP2-R190C in Normal CD8^+^ Cells Reduces the Effector Function

**Figure 4:**
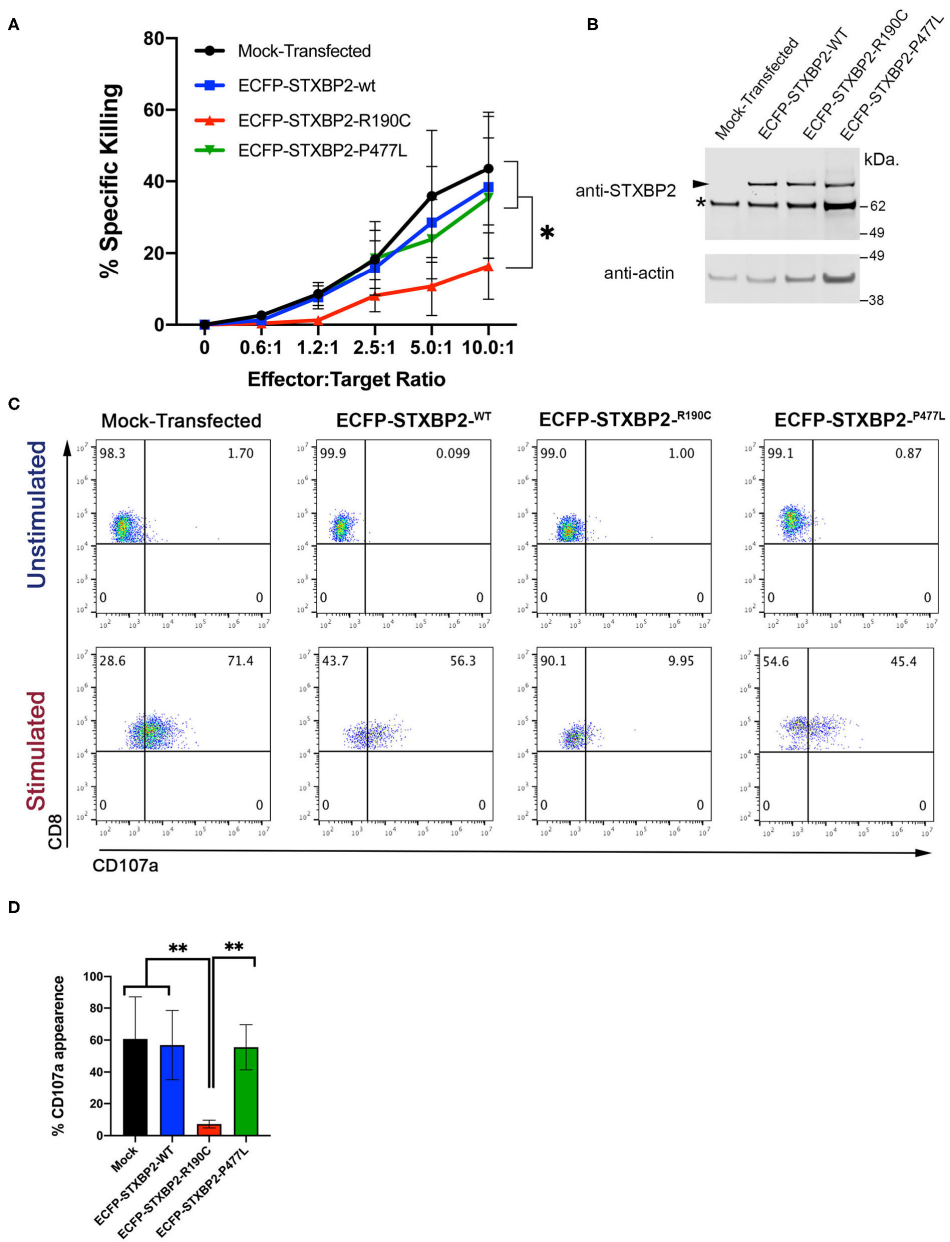
Expression of Munc18-2^R190C^ in control CTLs reduces cytolytic activity. **(A)** CD8^+^ T cells from H.C. donors were electroporated with ECFP-Munc18-2^R190C^, ECFP-Munc18-2^P477L^ ECFP-Munc18-2^WT^, or mock-transfected. Two days posttransfection, the cytotoxic activity against anti–CD3-coated P815 cells was tested at the indicated effector:target cell ratios. **(B)** Western blots of transfected cells developed with either anti-STXBP2 antibody (top panel) or anti-actin (lower panel). Arrow points at the transfected ECFP-STXBP2 protein; asterisk marks the position of endogenous STXBP2. **(C)** CD107a degranulation assay for ECFP-expressing cells. Transfected cells were incubated in the presence or absence of CD3-coated P815 cells for 4 h at 37°C. Cells were stained using anti–CD107a-PE, anti–CD8-FITC, and anti–CD3-PerCP antibodies and analyzed by flow cytometry. Plots are representative of two independent experiments. **(D)** Graph showing the percentage of cells that increased CD107a staining upon stimulation. Plots are mean ± SD of at least three independent experiments. Statistical analysis was done by one-way ANOVA **p < 0.01, *p < 0.05.

Because the STXBP2 R190C mutation identified in the subjects is monoallelic, and it does not affect the STXBP2 protein expression level or interferes with the interaction with STX11, we wanted to investigate whether it might act in a dominant-negative fashion as it was described for other STXBP2 mutations (14). To test this hypothesis, we analyzed whether the sole expression of STXBP2-R190C in H.C. CD8^+^ cells is sufficient to impair its effector functions. For this purpose, we transfected H.C. CD8^+^ cells with either ECFP-STXBP2-WT, ECFP-STXBP2-R190C, or ECFP-STXBP2-P477L or mock-transfected. Mutation STXBP2-P477L was previously described a pathogenic variant as a biallelic form only (9, 10). Analysis of cell killing activity against P815 target cells displayed that ECFP-STXBP2-R190C–transfected cells have a reduced activity, but not ECFP-STXBP2-WT, ECFP-STXBP2-P477L, or mock-transfected cells (Figure 4A). To evaluate whether the effect of ECFP-STXBP2-R190C was due to a significant overexpression of the mutant form, we compared the expression level of ECFP constructs with the endogenous STXBP2 protein by WB (Figure 4B). Results show that all ECFP-STXBP2 constructs were expressed at similar levels and were ~30% less than the endogenous STXBP2 protein. Evaluation of the CD107a degranulation showed that ECFP-STXBP2-R190C–transfected cells also have a reduced number of CD107a-positive cells compared with the -WT, -P477L, and mock-transfected (Figures 4C,D). Altogether, these results show that R190C mutation interferes with STXBP2 function and that the forced expression of STXBP2-R190C variant in normal CD8^+^ cells, which express endogenous STXBP2-WT, affects its degranulation and killing activity. Therefore, these results suggest that STXBP2-R190C might act in a dominant-negative fashion.

### STXBP2-R190C–Expressing CD8 Cells Fail to Induce Apoptosis in Target Cells

**Figure 5:**
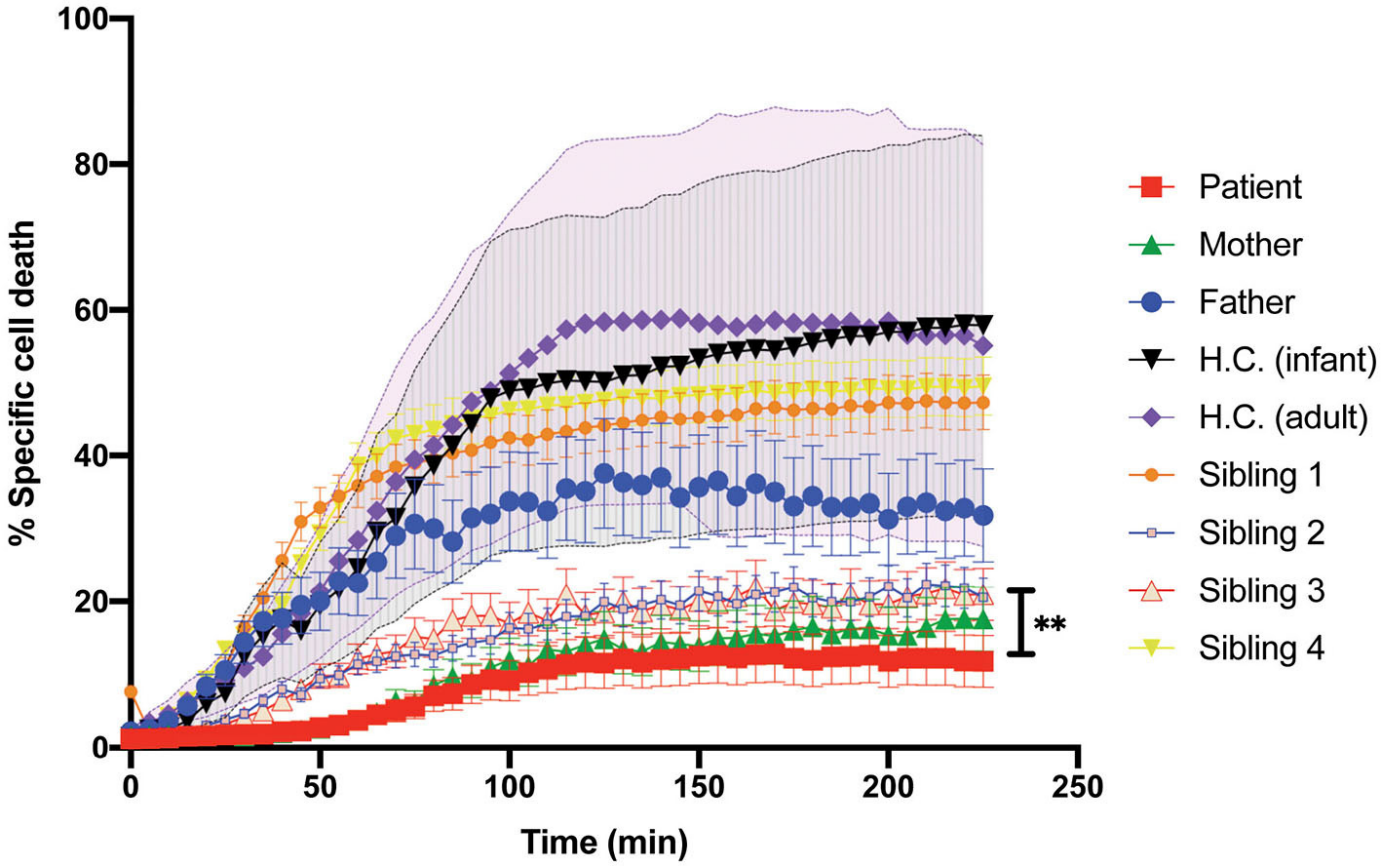
Killing activity of the patient CD8^+^ cells evaluated with the early-Tox caspase-3/7 assay. Target and effectors cells were incubated separately with a membrane-permeable NucView-488 caspase 3/7 substrate during 30 min. CD8^+^ cells from patient (red), mother (green), father (blue), and age-matched H.C. (infants: black; adults: purple) were activated with OKT3 15-min previous incubation with the substrate ends. Effectors and target cells were combined in 10–1 ratio in a black flat-bottom 96-well-plate. The green fluorescence signal was measured in each cell for 225 min with an interval of 5 min in a fluorescence cell imager ImageXpress Pico. The percentage of specific cell death was quantified as the percentage of cells that became positive for green fluorescence over time. H.C. range was established for both adults (pink shade) and infants (gray shade) from at least three to five different samples. Plots are mean ± SD of at least three independent experiments. Statistical analysis was done by one-way ANOVA **p < 0.01.

Patient's CD8^+^ cells expressing STXBP2-R190C displayed a severe defect, which is CD107a degranulation, which did not fully correspond with the partial reduction of their cytotoxic activity (20–30%) when measured using an end-point colorimetric LDH-release assays. To further investigate about the low correlation between both assays, we analyzed the capacity of the CD8^+^ cells to induce target-cell apoptosis by using a more sensitive and specific kinetic assay that monitors the activation of caspases-3 and−7 in single target cells over time. To this end, target cells loaded with a membrane-permeable NucView-488 caspase 3/7 substrate were incubated in the presence or absence of CD8^+^ cells. Upon caspase 3/7 activation in target cells, the substrate is cleaved and becomes fluorescent, and the number of positive green fluorescent cells was monitored over time by using an automatic cell imager as described in *Materials and Methods*. Results showed that patient, mother, sibling 2, and sibling 3 CD8^+^ cells exhibit a significant reduction in both speed and extent to induce caspase 3/7 activation (~50%, Figure 5) when compared with activity of the father, sibling 1, sibling 4, and corresponding age-matched H.C. Therefore, our results demonstrate that CD8^+^ cells from individual carrying STXBP2-R190C mutation display a profound defect in their ability to degranulate and to induce target-cell apoptosis.

## Discussion

Here we characterized the functional and biological relevance of a maternally inherited heterozygous variant *STXBP2* (c.568C>T; p.Arg190Cys) found in a patient manifesting with neonatal HLH and compounded with G6PD deficiency. Consistent with HLH diagnostic, our results showed that the degranulation ability of CD8^+^ and NK cells of the patient was significantly reduced. Interestingly, CD8^+^ and NK cells of the mother, sibling 2, and sibling 3, who also carry the STXBP2-R190C variant, also displayed impaired degranulation. We found that STXBP2, STX11, RAB27a, and Munc13-4 protein expression levels in the patient PBMCs were comparable to those of H.C. cells. Moreover, we found that endogenous STX11 coimmunoprecipitated to the same extent with STXBP2 in patient cells than H.C. Therefore, mutation R190C does not interfere with protein stability neither with the interaction with STX11. This phenotype closely resembled that observed in cells from f-HLH patient expressing STXBP2-R65Q (14) rather than in other patients having mutations that resulted in decreased STXBP2 and STX11 protein levels such as P477L (9) and G541S (16).

To better understand how this mutation might interfere with cytolytic function, we investigated whether any of the predicted functions of STXBP2 were affected by the R190C mutation, including its role as a chaperone or as an activator of membrane fusion. STX11 is one of the main binding partners of STXBP2 in immune cells (9, 10, 16, 22, 23) and platelets (30, 31). However, additional SNARE proteins have also been described to interact with STXBP2 in other cell types (32–34). To evaluate the putative chaperone function of STXBP2, we tested the subcellular distribution of EYFP-STX11 in control CTLs cotransfected with either ECFP-STXBP2-WT or ECFP-STXBP2R190C. These experiments strongly suggest that R190C mutation has no significant effect on the chaperone function of STXBP2 and correlates with our immunoprecipitation experiments, which showed a normal interaction between STXBP2 R190C and STX11. To evaluate the effect of R190C mutation on membrane fusion, we transfected ECFP-STXBP2-R190C, ECFP-STXBP2-P477L, or ECFP-STXBP2-WT in normal CD8^+^ cells. The expression of ECFP-STXBP2-R190C, but not of ECFP-STXBP2-P477L or ECFP-STXBP2-WT, was sufficient to reduce the cytotoxicity and degranulation activity. Because ECFP-STXBP2-R190C does not seem to be overexpressed compared with the endogenous STXBP2-WT, our results suggest that ECFP-STXBP2-R190C can outcompete STXBP2-WT and might act in dominant-negative fashion as it was previously described for other f-HLH variants (14, 35).

**Figure 6:**
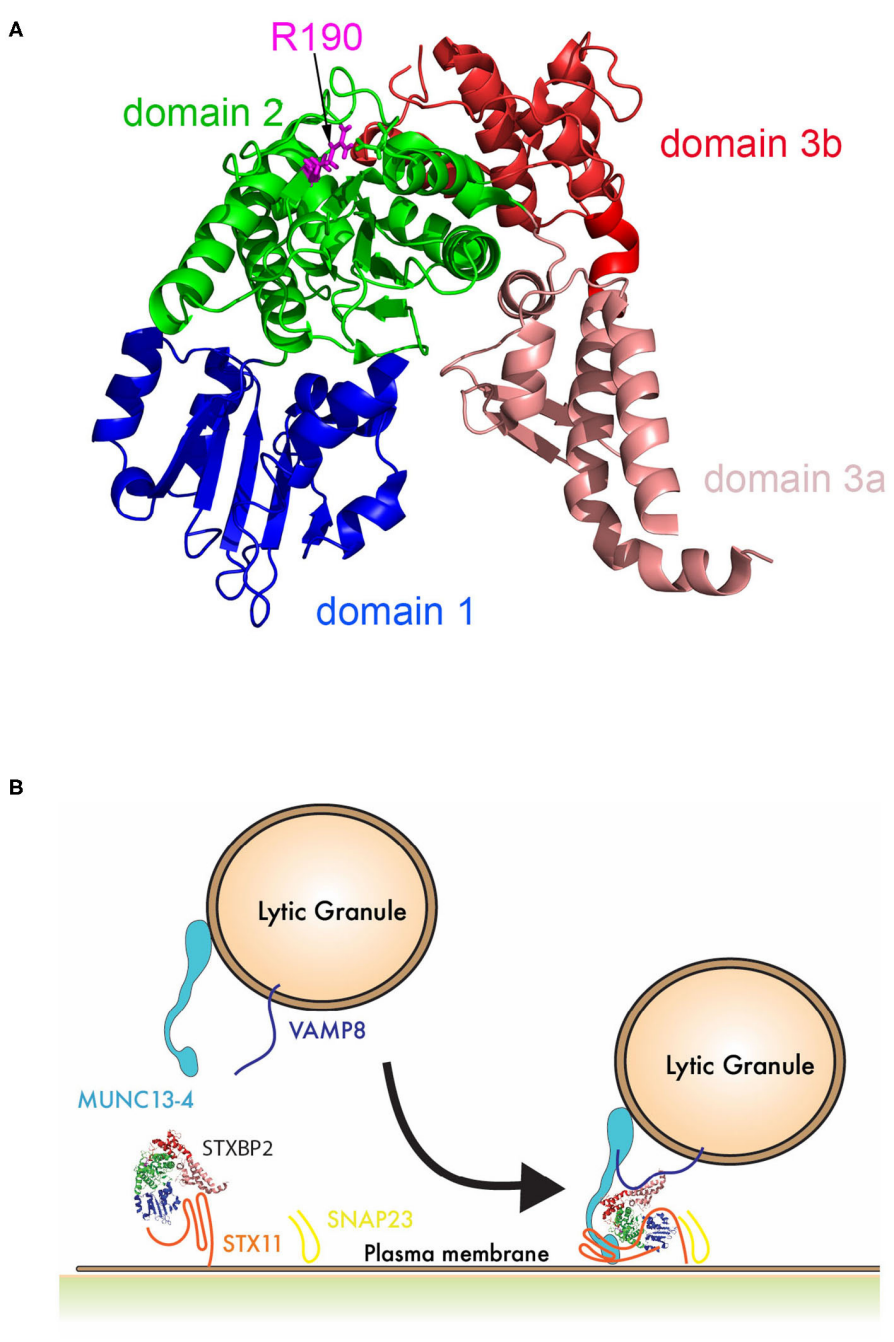
Schematic representation of the role of STXBP2 domain 2 on membrane fusion. **(A)** Crystal structure of Munc18-2 (PDB:4CCA) showing the different domains of STXBP2 based on STXBP1 crystal structure (45): domain 1 (blue) comprises residues 4–134, domain 2 (green) residues 135–245 and from 477–590, domain 3a (pale red) residues 246–361, and domain 3b (red) residues 362–476. **(B)** STXBP2 stabilizes STX11 in a closed conformation using domain 1 and 3a; upon the vesicle approximates, the immunological synapse membrane Munc13-4 may facilitate the transition of STX11 to an open conformation, allowing its interaction with domain 1 of STXBP2; VAMP can interact domain 3a of STXBP2, while domain 2 of STXBP2 stabilizes Munc13-4 in the complex.

Interestingly, upon further review of *STXBP2* mutations identified in other institutions, we found that this mutation is not unique to our patient, siblings, and his mother. This variant was previously reported in a 14-year-old HLH patient also carrying a heterozygous mutation in *STX11* (c.9C>A; p.Asp3Glu) (15). Moreover, arginine 190 is located in a structurally conserved region of domain 2 of STXBP proteins (Figure 6A). This region may play an important role in membrane fusion because mutations in neighboring residues, I232 and V487, also have been associated with f-HLH. Because the interaction between STX11/STXBP2 is not affected and it does not seem to directly involve this region, these data may reveal a novel binding mode of the STX11/STXBP2 complex with other factors, e.g., the surface of lipid bilayer or Munc13-4 protein, which is critical for membrane fusion to occur (Figure 6B). Consistent with this, *in vitro* studies using neuronal homolog proteins showed that MUN domains of Munc13-1 can interact with STX1/STXBP1 and induce a conformational change, allowing STXBP1 to serve as a scaffold protein and recruit VAMP2 through an interaction with STXBP1 domain 3a and thus promoting membrane fusion (36–39). Similarly, previous studies using yeast homolog proteins have shown that VPS33 (Sec/Munc) protein serves as scaffolding for SNARE-complex formation using domain 1 to interact with Vam3 (STX homolog) and domain 3a to interact with Nyv1 (VAMP homolog) (40). Nonetheless, how domain 2 of STXBPs contribute to these interactions remains to be investigated.

In addition to the *STXBP2* (c.568C>T; Arg190Cys) mutation, the patient also carries a mutation associated with G6PD deficiency. He met 6 of the 8 clinical criteria for HLH: splenomegaly, fever, hypertriglyceridemia, hemophagocytosis, extremely elevated ferritin, and elevated soluble IL-2 receptor (1). Liver dysfunction findings and the abnormal CD107a degranulation assay further supported the neonatal HLH diagnosis. Clinically, he responded to the HLH therapy according to HLH-2004 with resolution of the HSM, liver dysfunction, and declining ferritin. The association of HLH with significant involvement of liver dysfunction is often observed (41). However, the contribution of G6PD deficiency to HLH is uncertain; only two cases have been reported; parvovirus-B19–induced HLH in a 12-year-old with G6PD deficiency (42) and a 54-year-old with HLH and G6PD deficiency (43). In both cases, genetic workup was not reported. There was only mild hemolysis at the time of HLH diagnosis; however, G6PD deficiency could have aggravated the hyperbilirubinemia at birth. Interestingly, only after initiating CsA he developed significant anemia that required pRBC transfusions. This may have been CsA related eryptosis—the suicidal death of erythrocytes (44). Therefore, the extent by which G6PD deficiency has contributed to HLH in our patient still remains unclear.

Intriguingly, despite the severely impaired cytolytic function of CD8 and NK cells of the patient, sibling 2, sibling 3, mother, and a previously described patient (15), only our patient and the reported case in the literature clinically developed HLH. Our data suggest that the STXBP2-R190C variant has a high biological impact with a low clinical penetrance. Consistently, there are 91 presumptive healthy individuals in gnomAD v2.1.1 (https://gnomad.broadinstitute.org/variant/19-7706729-C-T?dataset=gnomad_r2_1) carrying the STXBP2-R190C variant. However, the cytolytic activity of these individuals still remains to be investigated. Usually, not all the individuals carrying a heterozygous mutation in the known HLH-associated gene clinically develop HLH. The wide spectrum of clinical symptoms and laboratory tests in these individuals, as it is in those carrying the variant STXBP2-R190C, could be due to differences in the penetrance of the allele in different individual, other unknown genetic modifiers, environmental factors, and/or to the exposure to the right challenge (e.g., specific virus) that could trigger the HLH. With our current genetic analysis, we could not completely rule out the possibility that another still unidentified gene variant might have compounded the effect of STXBP2-R190C in our patient and contributed for triggering HLH. For example, a yet unidentified variant inherited from the father, which caused a reduction of ~50% of the cytolytic function compared with normal controls (Figure 5), might have triggered the HLH manifestation by a digenic combined pathogenicity.

Taken together, these evidences further support the notion that mutation STXBP2-R190C similar to other heterozygous mutations in molecules involved in granule exocytosis can impact on CD8 and NK cell cytotoxic functions and might have a synergistic effect with other gene variants that ultimately could lead to HLH manifestation (12, 15).

## Data Availability Statement

The original contributions presented in the study are included in the article/supplementary material; further inquiries can be directed to the corresponding author/s.

## Ethics Statement

The studies involving human participants were reviewed and approved by The Institutional Review Board at Minnesota Children's Hospital. Written informed consent to participate in this study was provided by the participants' legal guardian/next of kin. Written informed consent was obtained from the individual(s), and minor(s)' legal guardian/next of kin, for the publication of any potentially identifiable images or data included in this article.

## Author Contributions

NB, WS, and MS performed experiments, collected, analyze, and discussed data. ES and MM generated reagents and performed experiments. YM developed the case report, collected the data, and wrote the manuscript. MV provided the genetic data and wrote the genetic elements of the manuscript. ML managed the patient and provided clinical and laboratory data. NE provided and wrote the hepatic elements of the manuscript. TP provided all immunologic studies and helped write the manuscript. CG performed experiments, analyzed and interpreted data, and wrote manuscript. All authors contributed to the article and approved the submitted version.

## Conflict of Interest

The authors declare that the research was conducted in the absence of any commercial or financial relationships that could be construed as a potential conflict of interest.
